# Drivers of intraspecific genetic differentiation of a wheat’s wild relative *Leymus racemosus*: roles of isolation by distance and environmental factors

**DOI:** 10.3389/fpls.2025.1675027

**Published:** 2025-11-18

**Authors:** Zhuo Zhang, Xiaojun Shi, Haowen Tian, Dunyan Tan

**Affiliations:** 1 Xinjiang Key Laboratory for Ecological Adaptation and Evolution of Extreme Environment Organisms, College of Life Sciences, Xinjiang Agricultural University, Urumqi, Xinjiang, China; 2 State Key Laboratory of Ecological Safety and Sustainable Development in Arid Lands, Xinjiang Institute of Ecology and Geography, Chinese Academy of Sciences, Urumqi, China

**Keywords:** *Leymus racemosus*, dd-RAD, genetic diversity, demographic history, environmental factors

## Abstract

Understanding how environmental heterogeneity shape genetic structure is fundamental to unraveling adaptive evolution and guiding conservation practices. Northern Xinjiang, characterized by its unique geological history and complex ecosystems, provides an ideal system to explore species-environment interactions. The riverine system, topographic relief, arid climate, and other factors have collectively shaped the genetic structure and adaptive trajectories of regional species. *Leymus racemosus*, a species well adapted to drought conditions, is primarily distributed along the Irtysh River basin. This study aims to examine the relative contributions of geographic distance, environmental factors, and their interactions to the genetic divergence of *L. racemosus*. Genomic SNP data from 135 individuals across 27 populations were generated through Double-digest restriction site-associated DNA (dd-RAD) sequencing. Population structure analyses employing ADMIXTURE, PCA, and phylogenetics revealed distinct genetic clusters. Gradient forest (GF) and redundancy analysis (RDA) were conducted to assess the environmental effects on genetic variation. Results suggest that low population genetic diversity and population structure analyses identified two evolutionarily distinct lineages (A and U). The divergence time between these two lineages can be traced back to the mid-to-late Last Glacial Period (0.0295 million years ago), a timescale that is highly consistent with the repeated glacial advances and retreats driven by the intense climatic fluctuations of the Quaternary Ice Age. Environmental association analyses (EAA) revealed significant correlations between allelic variation and climatic gradients, particularly with annual precipitation (bio12) and minimum temperature in the coldest month (bio6). Analyses of isolation by distance (IBD) and isolation by environment (IBE) further underscore the role of geographic distance and environment distance in the genetic differentiation of *L. racemosus*. This study reveals the pattern of genetic structure of *L. racemosus* populations and its association with environmental factors, contributing to the development of targeted strategies for species conservation and ecological restoration.

## 1 Introduction

Revealing the driving factors and processes that shape genetic variation in species is of significant interest to molecular ecology, evolutionary biology, and conservation biology (Dorogina and Zhmud, 2020; DeWoody et al., 2021; Kirschner et al., 2023; Walter et al., 2023; Wang and Qiu, 2024). The complex interplay of natural selection, gene flow, and genetic drift results in the formation of distinct spatial genetic structures within species (Eckert et al., 2008). Within this evolutionary framework, geography and environment act as two key determinants that mediate these processes, thereby influencing species-level genetic structure (Buiteveld et al., 2007). The Isolation-by-Distance (IBD) model posits that geographic distance and physical barriers restrict gene flow, leading to the local accumulation of genetic variants, such that genetic divergence increases with geographic separation (Wright, 1943; Orsini et al., 2013; Chang et al., 2022). In contrast, the Isolation-by-Environment (IBE) theory proposes that divergent natural selection driven by environmental heterogeneity constrains gene flow, resulting in positive associations between genetic differentiation and heterogeneous environments (Wang and Summers, 2010; Sexton et al., 2014; Wang and Bradburd, 2014; Jiang et al., 2019). Empirical evidence from multiple studies indicates that both IBD and IBE patterns are often simultaneously present within natural populations of the same species (Chang et al., 2022; Tumpa et al., 2022; Liu et al., 2023). Unraveling the relative importance of geography and environment in shaping patterns of genetic variation can provide deeper insights into how landscape factors influence evolutionary processes at both intra- and interspecific levels (De Kort et al., 2021).

The unique mountain-basin-desert-oasis geomorphology of Xinjiang, China, has been shaped by the combined effects of the uplift of the Qinghai-Tibet Plateau and Quaternary aridification, along with other geological and climatic processes (Liu, 2004; Zhang et al., 2022). In northern Xinjiang, the interplay between hydrologic networks, paleogeological dynamics, and extreme aridity has profoundly influenced the genetic structure and evolutionary trajectories of various species (Ju et al., 2015; Yang et al., 2016; Gao, 2017; Jiang et al., 2019; Xue et al., 2020). This region encompasses the interconnected Irtysh-Ulungur dual-basin system, a biogeographic nexus where drainage patterns and historical climate fluctuations have left measurable genomic imprints. Research on local taxa, including *Leuciscus leuciscus baicalensis* (Yang et al., 2017) and *Esox lucius* (Luan et al., 2021), reveals genetic divergence patterns that are associated with Pleistocene glacial cycles in the Altai Mountains. Additionally, *Populus alba* populations in the Irtysh basin exhibit low-frequency polymorphisms that are consistent with postglacial recolonization following a genetic bottleneck (Liu et al., 2019a). These findings provide critical insights into how climate and environmental change have shaped genetic differentiation in the different taxonomic groups of flora and fauna in this arid land basin system.

The genus *Leymus* (Poaceae),with its exceptional salt and drought tolerance, plays vital roles in ecological restoration and grassland management across Eurasian steppes (Su et al., 2013). The Altai region of Xinjiang harbors the highest species diversity of *Leymus*, which were considered as a primary diversification center for the genus (Wu, 1979; Zhi and Teng, 2005; Liu et al., 2017a). Current evidence indicates significant interspecific genetic divergence within *Leymus*, while intraspecific populations exhibit high genetic homogeneity and close phylogenetic relationships (Yang et al., 2006). Notably, populations of *Leymus chinensis* across China display east-west genetic clines, with significantly higher diversity observed in northeastern regions compared to the western arid zones (Wang et al., 2005; Yuan et al., 2016). However, habitat patch size and environmental heterogeneity strongly influence the genetic structure of *L. chinensis* (Liu et al., 2004; Guo et al., 2021). In conclusion, research about the genetic of *Leymus* is mainly focused on the specific species *L. chinensis*, while the genetic diversity of other *Leymus* plants is insufficient, limiting our further development and application of *Leymus* resources.

**Figure 1:**
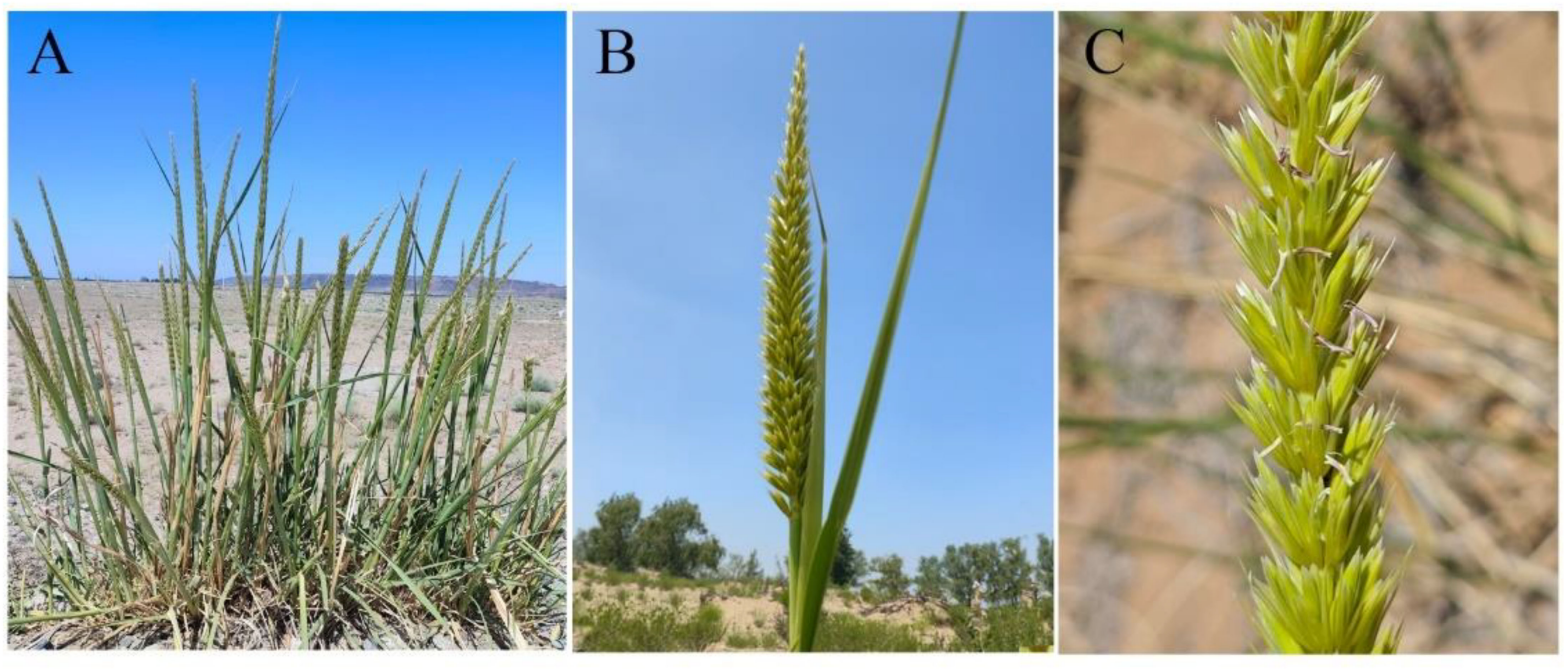
Morphological characteristics of *Leymus racemosus*. **(A)** plant; **(B)** spike; **(C)** spikelet.

*Leymus racemosus* (Lam.) Tzvelev, a member of *Leymus*, is a perennial grass endemic to the mobile dunes and sandy steppes of China, Russia and Central Asia. In China, it is exclusively found in northern Xinjiang, where it primarily inhabits stabilized or semi-stabilized dunes along the Irtysh River (Wu, 1996). This species exhibits distinctive morphological adaptations, including large panicles, sturdy stems, and extensive rhizome networks (**Figure 1**), which enhance dune stabilization by increasing surface roughness and effectively mitigating wind erosion (Dong et al., 1985; Huang et al., 1997). As a wild relative of wheat (Triticeae), *L. racemosus* is tolerant to salt and drought and resistant to various diseases, such as *Fusarium graminearum* (Wang and Zhang, 2019), providing excellent genetic resources for wheat improvement (Edet et al., 2018). Therefore, understanding its population genetic structure and genotype-environment interactions is essential for effective utilization of these genetic resources, guiding future breeding efforts to develop varieties that are resilient to climate change and other environmental stressors.

In recent years, research on *L. racemosus* has predominantly focused on various aspects, including wheat breeding, karyotype studies, mating systems, seed characteristics, fruiting patterns, and genetic diversity (Liu and Zhuo, 2008; Holderegger et al., 2010; Gulnar et al., 2014; Hu et al., 2014; Xue et al., 2015). However, the genetic variation data obtained from previous studies utilizing molecular markers such as ISSR and AFLP are relatively limited. Crucially, no studies to date have integrated environmental data with high-throughput genomic markers to unravel genotype-environment associations in this species. Particularly in the realm of adaptive evolution in response to environmental changes, relevant studies remain sparse. In the context of climate change and ecosystem degradation, research on the genetic structure of *L. racemosus* populations and their associations with environmental variables is still insufficient. As this species exhibits a fragmented population pattern across its range, investigating the relationship between genetic diversity and landscape features is of considerable theoretical importance for elucidating the species’ adaptive influencing factors and optimizing conservation strategies. The objectives of this study were to: (1) assess the genetic diversity and genetic structure of *L. racemosus*; (2) estimate the timing and pattern of divergence among populations of *L. racemosus*; and (3) investigate the roles of geographic distance and environmental factors on genetic variation of *L. racemosus*.

## 2 Materials and methods

### 2.1 Sample collection and DNA extraction

**Figure 2:**
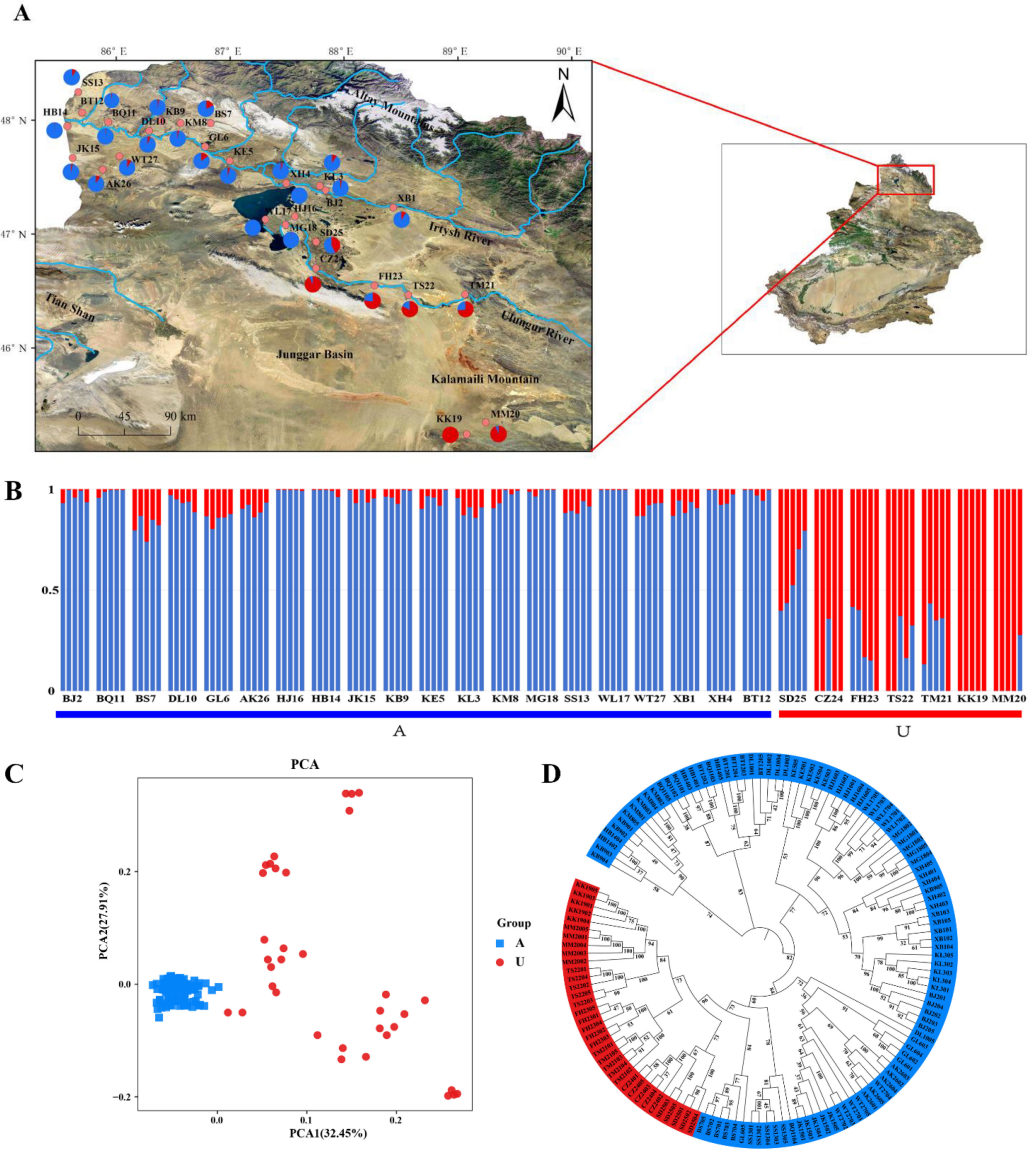
Genetic structure analysis of 27 populations and 135 individuals of *Leymus racemosus*. **(A)**, The geographical locations of 27 *Leymus racemosus* populations. The pie chart shows the average proportion of the two genetic lineages identified in each group. Blue represents lineage A and red represents lineage U. **(B)**, ADMIXTURE phylogenetic typing. The blue and red bars represent the likelihood Q values of lineages A and U respectively. **(C)**, Principal component analysis (PCA). **(D)**, Neighbor-Joining (NJ) phylogenetic tree.

A total of 135 *L. racemosus* individuals were sampled from 27 geographically distinct populations in northern Xinjiang of China (**Figure 2A**; **Supplementary Table S1).** To minimize clonal sampling, individuals within each population were spaced at least 10 m apart. Fresh leaf tissues from healthy plants per individual were collected and immediately desiccated using indicating silica gel, followed by long-term storage at -80°C. Genomic DNA was extracted using a modified CTAB protocol (Porebski et al., 1997) and processed by Shanghai Personal Biotechnology Co., Ltd. The quality of the DNA was verified through 0.8% agarose gel electrophoresis and spectrophotometry.

### 2.2 Genome sequencing and SNP filtering

Double-digest restriction site-associated DNA (dd-RAD) libraries were constructed using *HindIII* and *MspI* (New England Biolabs), targeting size selection of 300–500 bp fragments. The libraries were quantified with an Agilent High Sensitivity DNA Kit and a Quant-iT PicoGreen dsDNA Assay Kit, followed by paired-end sequencing (2×150 bp) on an Illumina NovaSeq 6000 platform. Approximately 34,971,347 raw reads per sample were generated, with Q30 scores exceeding 95% (**Supplementary Table S2**). Raw reads were processed using fastp (https://github.com/OpenGene/fastp) for adapter trimming and quality filtering via a sliding window approach (5 bp window), removing reads with more than 10% ambiguous bases or adapter contamination. Clean reads were aligned to the *L. chinensis* reference genome (https://doi.org/10.6084/m9.figshare.24032238.v1) using BWA-MEM (Li and Durbin, 2009), SNP calling was performed with GATK and filtered using VCFtools (Danecek et al., 2011; Zhu et al., 2014) with the following thresholds: Quality Depth (QD) <2.0; Fisher’s Strand Bias (FS) >60.0; Mapping Quality (MQ) <40.0; minor allele frequency (MAF) >0.05; maximum missing data per site ≤20%; read depth (DP) between 2 and 1,000; and linkage disequilibrium (r²) <0.2 in 50 bp sliding windows with 5 bp steps. This stringent filtering resulted in the retention of 56,655 high-confidence SNPs for downstream analyses.

### 2.3 Genetic diversity analysis.

Genetic diversity metrics were calculated for the filtered SNP dataset using PLINK (Purcell et al., 2007). Observed heterozygosity (*H_O_*), expected heterozygosity (*H_E_*), and the inbreeding coefficient (*F_IS_*) were estimated to quantify genetic variation at the population level. Values of *F_IS_* greater than 0 indicate an excess of homozygosity (inbreeding), while values less than 0 suggest a surplus of heterozygotes relative to Hardy-Weinberg expectations. Population differentiation was assessed through pairwise *F_ST_* estimates using VCFtools, with interpretations as follows: 0-0.05 (negligible differentiation), 0.05-0.15 (moderate differentiation), and greater than 0.15 (strong differentiation). Neutrality tests were conducted by calculating Tajima’s *D* across sliding windows (5 kb window, 10 kb step), where significantly positive values indicate balancing selection or population contraction, while negative values suggest directional selection or population expansion. Nucleotide diversity (*P_i_*) was computed as the average pairwise differences per site, with lower values (<0.005) reflecting reduced genetic variation within populations.

### 2.4 Population genetic structure analysis

Use VCFtools and Plink software successively to convert files into plink format and the “bed” format required by admixture. Subsequently, ADMIXTURE (Patterson et al., 2012) is employed to construct the genetic structure, with the range of genetic clustering values (*K*) set from 2 to 10, utilizing the software’s default parameters. The optimal K value is defined as the one with the smallest cross-validation (CV) error value. The population structure diagram is generated using the R language (v4.1.0) and the Pophelper package. Additionally, we conducted Principal Component Analysis (PCA) on the samples using PLINK software to further analyze the population’s genetic structure. To elucidate the evolutionary relationships and genetic differences among 135 individuals, we performed Neighbor-Joining (NJ) tree-building analysis using MEGA(v11.0.13) software (Tamura et al., 2021). The phylogenetic tree was constructed using the Kimura 2-parameter model, with bootstrap support assessed from 1,000 replicates. All statistical analyses adhered strictly to the usage specifications and parameter settings of the respective software to ensure the reproducibility and scientific integrity of the results.

### 2.5 Population evolutionary history

To facilitate the analysis of population dynamic history using DIY ABC software (Cornuet et al., 2010), we further filtered SNPs using VCFtools at intervals of 300 kb, resulting in a total of 12,700 high-quality SNPs. Based on Approximate Bayesian Computation (ABC) at a 95% confidence level, combined with logistic regression, we estimated the posterior probability (PP) of each evolutionary event. The evolutionary event with the highest posterior probability was identified as the optimal evolutionary event. The results of the DIYABC calculations expressed intergroup differentiation in terms of generations. To determine the timing of intergroup differentiation, it is essential to multiply the generation time of the species. For perennial herbaceous plants, accurately determining their generation time is often challenging. Consequently, this study calculated the divergence time based on the generation time of the closely related species *L. chinensis*, which is 2 years.

### 2.6 Environmental association analysis

To investigate the impact of climatic and soil factors on the genetic variation of *Leymus racemosus* populations, 19 bioclimatic variable layers were downloaded from the PaleoClim database (http://www.PaleoClim.org/) and climate data for each population were extracted using the raster package in R. Using ArcGIS v10.8 based on the longitude and latitude of the sampling points, 15 soil factor data were extracted from the Harmonized World Soil Database (HWSD). All climatic and soil data underwent preprocessing. To mitigate multicollinearity issues, Pearson correlation analysis (Cleophas and Zwinderman, 2018) was conducted on the climatic and soil data. Variables with a correlation coefficient (*r*) greater than 0.8 were removed, and the contribution rate ranking was assessed using the maximum entropy model algorithm. Ultimately, 8 climate factors and 8 soil factors with high contribution rates and no overfitting were selected for subsequent Gradient Forest Analysis (GF) and Redundancy Analysis (RDA) (**Supplementary Table S3)** (Ellis et al., 2012; Capblancq and Forester, 2021).

The Mantel test was employed to assess potential patterns of Isolation by Distance (IBD) and Isolation by Environment (IBE) in *Leymus racemosus* populations, evaluating the correlation between genetic distance and both geographical and environmental distances. Genetic distance was calculated using the population differentiation index (*F_ST_*), with a linearized *F_ST_*/(1 - *F_ST_*) transformation applied to quantify genetic distance. The geographical distance between populations was determined using the R package geosphere (Karney, 2013). Eight climate factors and eight soil factors, extracted from the HWSD and PaleoClim databases, were utilized to compute the Euclidean distance matrix between the variables. The R package vegan was employed to conduct 1000 permutation tests (Dixon, 2003) on the genetic distance matrix and the geographical or environmental distance matrices, calculating the correlation coefficient (*r*) and its significance level (*p*-value). Scatter plots and trend lines depicting genetic distance versus geographical or environmental distance were generated to illustrate significant correlation patterns.

## 3 Results

### 3.1 SNP screening and population genetic diversity

After stringent quality control, a total of 56,655 SNPs were retained for downstream analyses. The genetic diversity analysis revealed that the inbreeding coefficient (Fis) ranged from 0.5810 to 0.7019, indicating a high degree of genetic similarity and low heterozygosity within the population (**Table 1**). This pattern is consistent with the species’ known clonal propagation strategy. The expected heterozygosity (*H_E_*) ranged from 0.1705 to 0.2171, while the observed heterozygosity (*H_O_*) ranged from 0.05901 to 0.0830. The heterozygosity of the *L. racemosus* population is significantly lower than the expected value, suggesting that factors such as inbreeding, population structure, or genetic drift may be present, resulting in heterozygosity that is lower than theoretical expectations and a reduction in genetic diversity. The *Pi* value ranged from 0.0353 to 0.0380, reflecting significant DNA sequence variation among individuals and a low level of genetic diversity. The Tajima’s *D* values (0.518-0.655) suggested potential balancing selection or a recent contraction of the population. Genetic differentiation metrics revealed that populations KK19 and MM20 exhibited pronounced genetic divergence from other populations, whereas populations TM21, TS22, FH23, CZ24 and SD25 displayed relatively low genetic differentiation, and minimal genetic divergence was observed among the remaining populations (**Supplementary Figure S1).**

**Table 1:** Genetic diversity indexes of the population of *Leymus racemosus*.

Population	Number	*F_IS_*	*H_O_*	*H_E_*	*P_i_*	Tajima’s *D*
XB1	5	0.6684	0.0708	0.2136	0.0377	0.5432
BJ2	5	0.6973	0.0693	0.2105	0.0369	0.5287
KL3	5	0.6697	0.0652	0.2101	0.0373	0.5318
XH4	5	0.5810	0.0718	0.2124	0.0357	0.6142
KE5	5	0.6675	0.0692	0.2090	0.0371	0.5183
GL6	5	0.6324	0.0662	0.2136	0.0363	0.5807
BS7	5	0.6875	0.0699	0.2131	0.0367	0.5411
KM8	5	0.7087	0.0676	0.2128	0.0366	0.5600
KB9	5	0.7036	0.0705	0.2162	0.0369	0.5556
DL10	5	0.6881	0.0712	0.2171	0.0371	0.5501
BQ11	5	0.6976	0.0641	0.2138	0.0363	0.5539
BT12	5	0.6709	0.0695	0.2158	0.0375	0.5249
SS13	5	0.6642	0.0648	0.2099	0.0374	0.5386
HB14	5	0.6880	0.0662	0.2149	0.0372	0.5470
JK15	5	0.6622	0.0630	0.2104	0.0375	0.5246
HJ16	5	0.6331	0.0613	0.2089	0.0380	0.5926
WL17	5	0.6793	0.0591	0.2011	0.0378	0.5800
MG18	5	0.6853	0.0628	0.2012	0.0375	0.5729
TM21	5	0.6640	0.0611	0.1826	0.0358	0.5751
TS22	5	0.6471	0.0620	0.1706	0.0360	0.5739
FH23	5	0.7019	0.0706	0.2132	0.0366	0.5866
CZ24	5	0.7017	0.0749	0.2146	0.0364	0.5616
SD25	5	0.6655	0.0746	0.2073	0.0375	0.5427
AK26	5	0.6595	0.0830	0.2036	0.0378	0.5285
WT27	5	0.6740	0.0723	0.2058	0.0374	0.5406
KK19	5	0.6885	0.0614	0.2117	0.0366	0.5466
MM20	5	0.6463	0.0623	0.2106	0.0353	0.6547

*H_O_*, observed heterozygosity; *H_E_*, expected heterozygosity; *F_IS_*, inbreeding coefficient; *P_i_*, Nucleotide diversity.

### 3.2 Population genetic structure

STRUCTURE analysis identified K = 2 as the optimal number of clusters using the cross-validation error (CV) method (**Figures 2A, B**; **Supplementary Figures S2**, **S3**). Lineage A consisted of 20 populations from the Irtysh basin, while lineage U included seven populations (KK19, MM20, TM21, TS22, FH23, CZ24, SD25) from the Ulungur basin and Kalamaili Mountains. Five populations (TM21, TS22, FH23, CZ24, SD25) exhibit varying degrees of genetic admixture, suggesting that these populations may occupy a transitional genetic zone between the lineages. Notably, the three populations near Ulungur Lake (HJ16, MG18, WL17) exhibited genetic proximity to those in the Irtysh River Basin. This observation is related to the geographical characteristics of Ulungur Lake, which serves as the connection point between the Irtysh River and the Ulungur River water systems. Principal Component Analysis (PCA) revealed that PC1 and PC2 accounted for 32.45% and 27.91% of the total genetic variation, respectively (**Figure 2C**). Individuals from distinct lineages exhibited significant genetic divergence, further corroborating the patterns identified in the STRUCTURE analysis. Phylogenetic tree analysis, supported by well-calibrated posterior probabilities, also confirmed this genetic structure (**Figure 2D**).

### 3.3 DIY-ABC-based inference of demographic history

**Figure 3:**
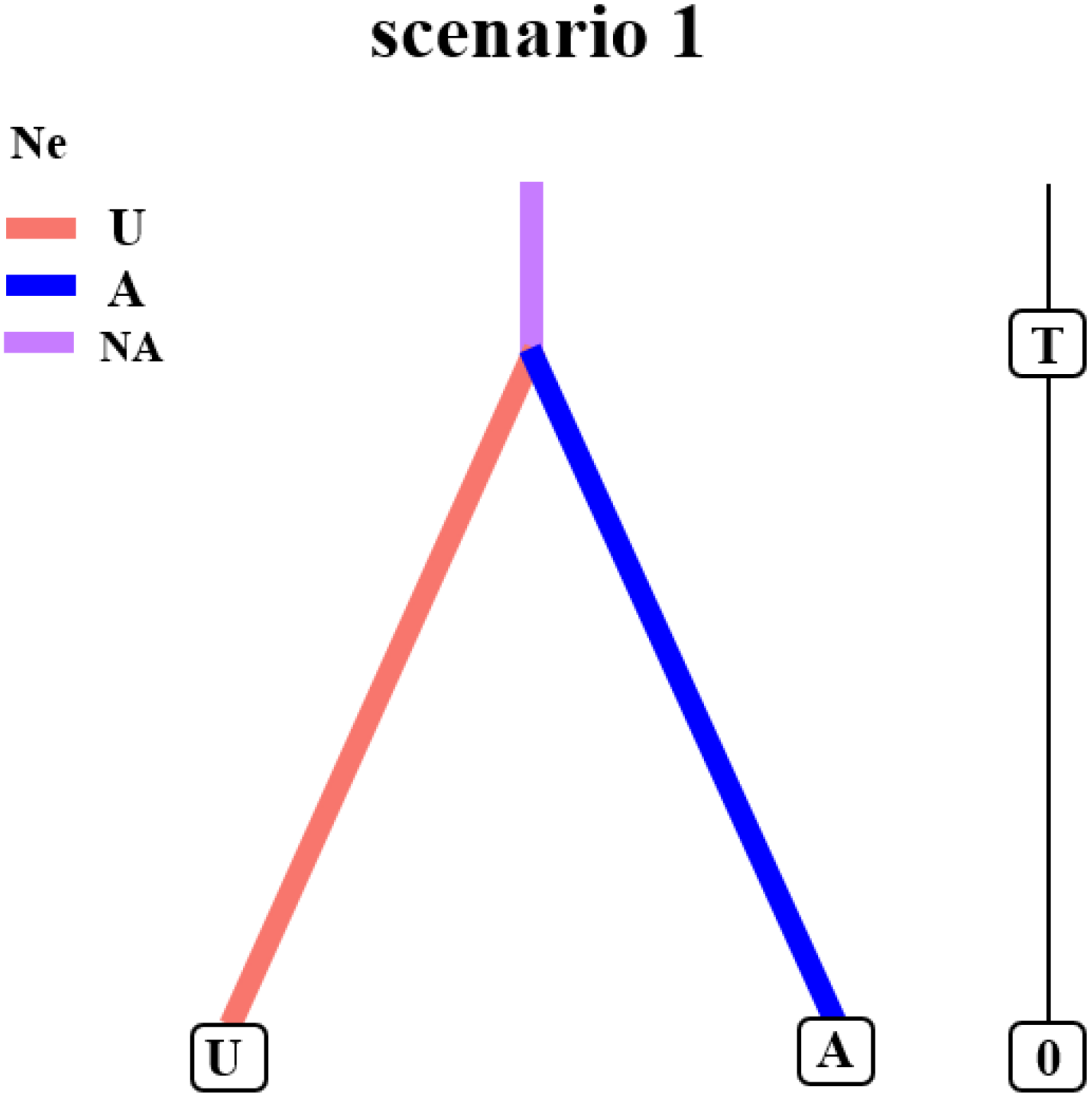
Population dynamics history of lineages U and A in *Leymus racemosus*.

We employed Approximate Bayesian Computation (ABC) as implemented in DIYABC v2.1.0 to evaluate three evolutionary scenarios for *L. racemosus*. Our analysis provides the strongest support for Model 1, which best explains the divergence of the A and U lineages (**Figure 3**; **Supplementary Figure S4**; **Supplementary Tables S4**, **S5**). The divergence time was estimated by scaling the expected value of the time parameter (t) by the generation time of *L. racemosus*. This analysis indicates that the A and U lineages diverged from a common ancestor approximately 0.0295 million years ago (Mya; 95% CI), during the mid-Pleistocene. These historical events have shaped the current phylogeographic structure, wherein the distribution of lineage U is centered in the Ulungur River basin, while lineage A is predominantly found in the Irtysh River basin.

### 3.4 Genetic and environmental association analysis

**Figure 4:**
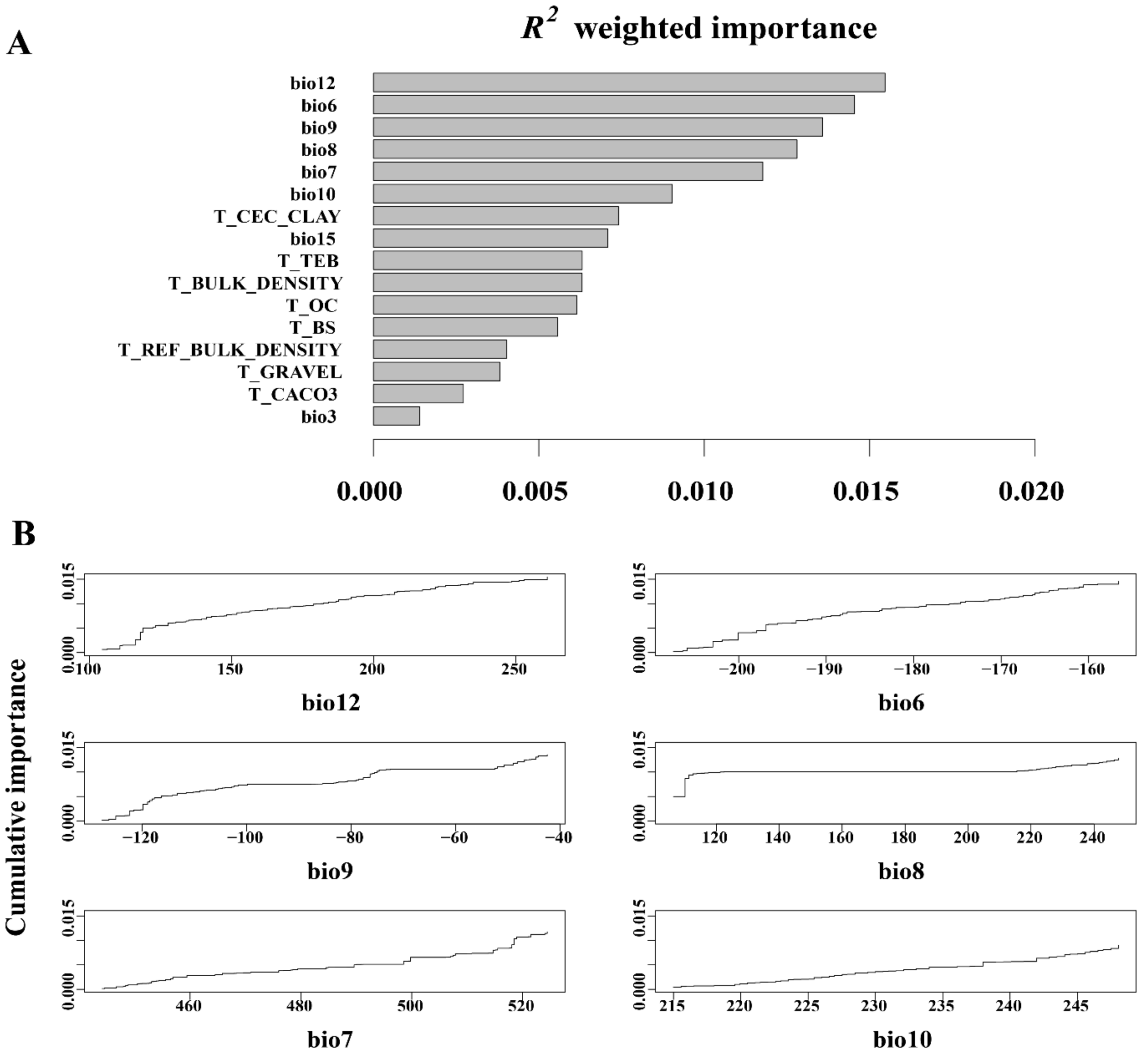
*Leymus racemosus* gradient forest (GF) analysis. **(A)**, *R2*-weighted importance of environmental variables that elucidate genetic gradients; **(B)**, The cumulative importance of allele variation in the first six environmental gradients.

Gradient forest analysis of 16 environmental predictors, comprising 8 climatic and 8 edaphic factors (**Figure 4**), revealed that bio12 (annual precipitation), bio6 (minimum temperature in the coldest month), bio9 (average temperature in the driest quarter), bio8 (average temperature in the wettest quarter), bio7 (annual temperature range), and bio10 (average temperature in the hot quarter) were identified as the six most significant climate factors influencing changes in allele frequencies within the *L. racemosus* population. The weights of the remaining 10 ecological factors were small, limited impact on genetic variation. Annual precipitation emerged as the most critical climatic factor, with its maximum weight underscoring the decisive role of water conditions in the growth and reproduction. Additionally, bio6, bio9, bio8, bio7, and bio10 suggest that temperature fluctuations significantly affect the physiological adaptability and genetic diversity. In contrast, the impact of soil factors on the genetic variation of *L. racemosus* appears to be relatively minor, likely due to soil homogeneity across the study area.

**Figure 5:**
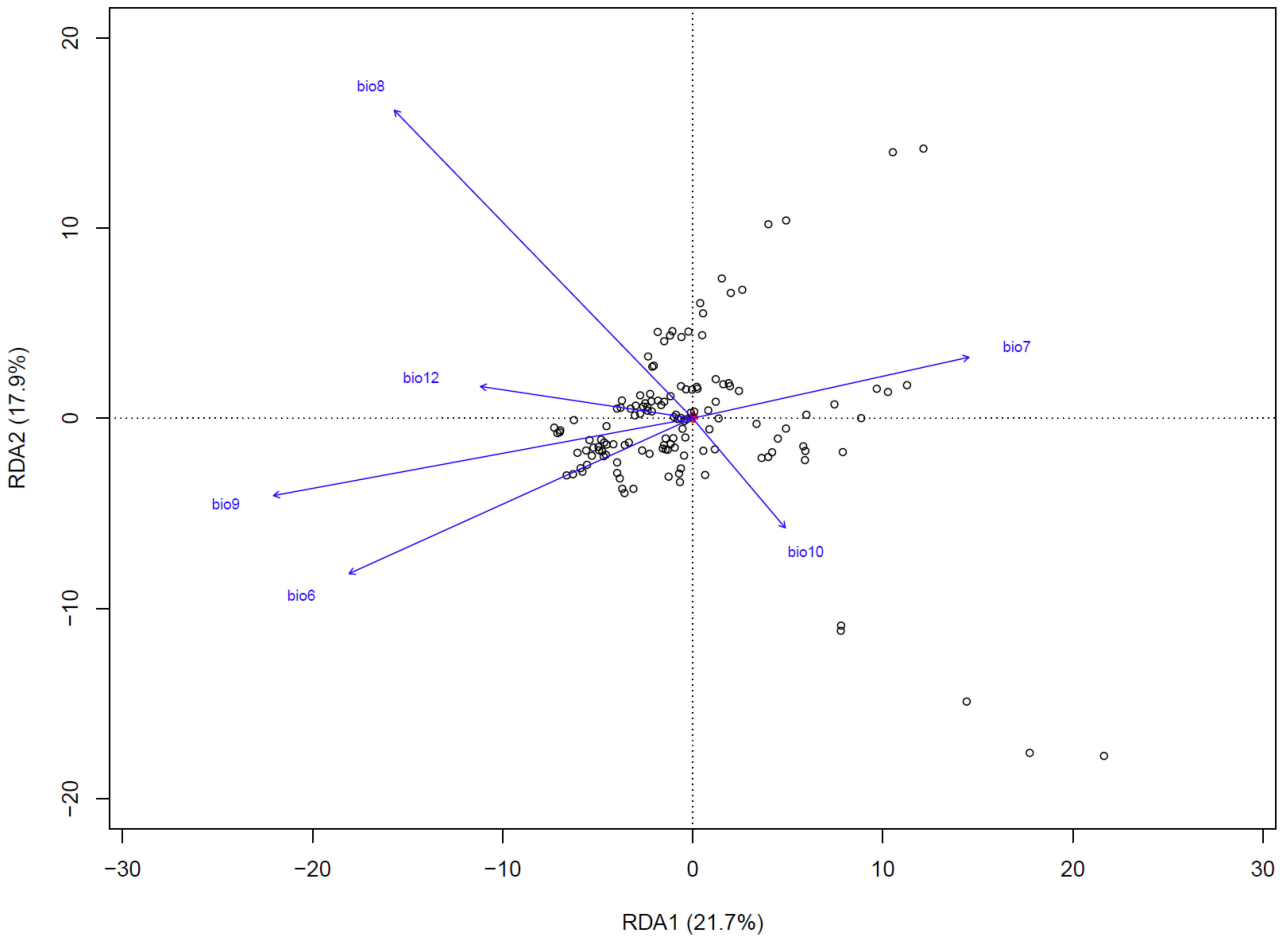
Redundancy analysis (RDA) of the relationship between 6 important environmental factors and genetic variation in *Leymus racemosus*. White circle, different individuals; small red points (located at the center of the figure), single nucleotide polymorphism (SNP) site.

**Figure 6:**
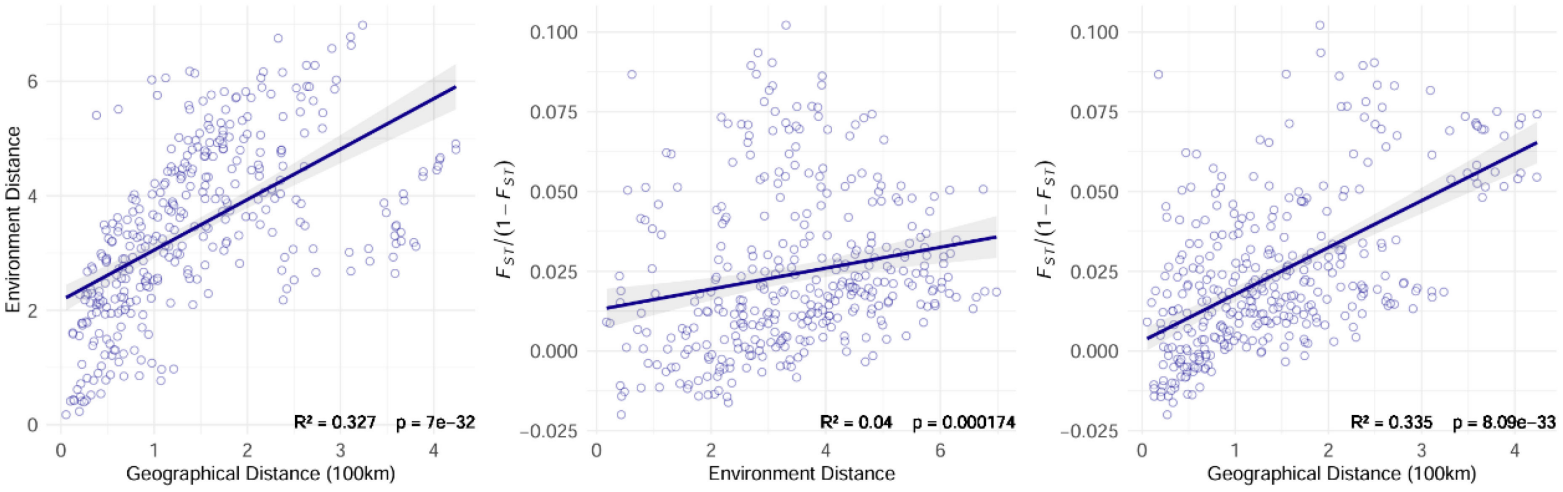
*Leymus racemosus* isolation by distance (IBD) and isolation by environment (IBE) analysis.

Redundancy analysis (RDA) revealed that climatic variables accounted for 39.6% of the total genetic variation, with RDA1 (21.7%) and RDA2 (17.9%) capturing the primary environmental-genetic covariation (**Figure 5**). Three thermal parameters exhibited the strongest loadings: the minimum temperature of the coldest month (bio6), the mean temperature of the wettest quarter (bio8), and the mean temperature of the driest quarter (bio9). These temperature extremes also emerged as the top predictors in gradient forest analysis, thereby confirming their dual roles as selective pressures and dispersal filters. Mantel tests quantified isolation patterns, revealing that genetic distance exhibited the strongest correlation with geographic distance (*R²* = 0.335, *p*<0.001) (**Figure 6**), followed by environment-geography covariance (*R²* = 0.327, *p*<0.001), while direct genetic-environment relationships were the weakest (*R²* = 0.04, *p*<0.001).

## 4 Discussion

### 4.1 The genetic structure of *L. racemosus* population and its geographical distribution pattern

The level of genetic diversity is closely linked to population reproductive strategies, as well as a species’ evolutionary potential and its ability to adapt to environmental changes (Jump and Peñuelas, 2005; Kim et al., 2019). In contrast to the high genetic diversity observed in its congeneric species, *Leymus chinensis* (*H_O_* = 0.780, *H_E_* = 0.916), we found that populations of *L. racemosus* exhibit relatively low genetic diversity (*H_O_* = 0.0674, *H_E_* = 0.2261). These results are comparable to those reported for other low genetic diversity species, such as *Albizia odoratissima* (*H_O_* = 0.189, *H_E_* = 0.189) and *Myricaria laxiflora* (*H_O_* = 0.1394, *H_E_* = 0.1468) (Li et al., 2025; Ou et al., 2025; Xiao et al., 2025). We attribute this low genetic diversity to the species’ facultative reproductive strategy. Under multiple environmental pressures, including drought stress, wind erosion, and human disturbance, the species exhibits reduced natural regeneration and lower seed set during the sexual reproduction phase (Wang et al., 2011; Gulnar et al., 2014). This has driven the species to evolve a survival strategy predominantly reliant on asexual reproduction. Although asexual reproduction enables rapid niche occupation, it leads to high genotypic homogeneity within populations, consequently reducing genetic diversity. Low genetic diversity is also observed in other species that have facultative reproduction, for example, *Scutellaria floridana* (Hanko et al., 2023), *Santalum lanceolatum* (Brunton et al., 2021), *Nypa fruticans* (Sugai et al., 2016), and *Santalum insulare* (Lhuillier et al., 2006). These similar genetic characteristics highlight the critical influence of reproductive modes on the genetic diversity levels of populations. Against the backdrop of low overall genetic diversity in the species, our results revealed that high genetic differentiation exists not only between lineages but also among certain populations (e.g., KK19 and MM20). We hypothesize that this differentiation is associated with the heterogeneity of their habitats: KK19 population is located on a severely wind-eroded, species-poor mountain ridge, while MM20 population is distributed across a relatively species-rich desert steppe. This pronounced difference in microhabitats is likely to be an important contributor to the strong genetic differentiation observed between these two populations (Xia et al., 2019). In addition, field surveys indicate that the *L. racemosus* population currently exhibits distinct patchy distribution characteristics. This phenomenon may be attributed to population isolation resulting from landscape fragmentation, which subsequently affects the genetic diversity and ecological adaptability of the population. Factors such as habitat loss, invasive species, water diversion, and alterations in river flow may drive this fragmentation (Rood et al., 2005; Thaulow et al., 2013; Mayonde et al., 2019; Pflueger et al., 2019), ultimately result in the loss of genetic connectivity at the landscape level, thereby causing a reduction in gene flow and change in the genetic diversity of species (Cushman et al., 2014). This phenomenon may further diminish genetic variation within populations, thereby compromising their long-term adaptive capacity.

Genome SNP analysis has revealed a significant phylogeographic structure within *L. racemosus*, which can be divided into two major lineages, designated A and U. The spatial distribution of these two lineages exhibits a clear pattern of river basin differentiation: lineage A is centered on the Irtysh River basin, whereas lineage U primarily occupies the Ulungur River basin, with gradual expansion into the Junggar Basin. This distribution pattern strongly suggests that lineage divergence within this species has been driven by a combination of geographical isolation and differential selection pressures (Liu et al., 2019b; Tóth et al., 2019). By integrating genetic structure analysis with geographical data, we found that the differentiation between lineages U and A may have been profoundly influenced by the regional river network. Within the Irtysh River basin, the genetic structure exhibits higher homogeneity, likely due to more extensive habitats and fewer geographical barriers, thereby promoting gene flow (Ma et al., 2016; Luo et al., 2023). In contrast, populations in the Ulungur River basin show signs of genetic admixture, which may reflect gene introgression during the differentiation process in this region, superimposed with the impacts of human activities (Mairal et al., 2022; Yan et al., 2024). Consequently, the two major basins, the Irtysh and Ulungur Rivers, can each act as corridors for gene exchange, but the area between these distinct basins constitutes a geographical barrier that promotes lineage differentiation. This dual role has been observed in a similar pattern in *Populus fremontii*, whereby small rivers and highlands restrict gene flow, whereas medium and large rivers facilitate genetic exchange (Cushman et al., 2014). Furthermore, the correlation between hydrological features—such as river gradient, confluence frequency, basin size, seasonal flow, and high-discharge events—and the spatial genetic structure further underscores the critical role of the riverine landscape in intraspecific lineage differentiation (Olsen et al., 2010; Cook et al., 2011; Kanno et al., 2011; Zhang and Sun, 2011). The complex interplay between hydrological connectivity and geographical isolation within these watersheds has fundamentally shaped the contemporary genetic architecture of *L. racemosus*.

### 4.2 The adaptation of *L. racemosus* to the pattern of natural landscape

This study investigates the adaptive evolution of *L. racemosus* in response to natural landscape patterns, with particular emphasis on how climate fluctuations and geomorphic changes have influenced population differentiation across various temporal and spatial scales (Bay et al., 2018; Du et al., 2020; Tian et al., 2024). Demographic analyses indicate that the initial divergence of *L. racemosus* populations occurred approximately 0.0295 million years ago. This timeframe corresponds to the mid-to-late phase of the Last Glacial Period (70-11.5 ka before present (BP)), suggesting that their divergence was driven by climatic fluctuations associated with Quaternary glaciation (Li et al., 2004). Since the Pleistocene, the continuous aridification of Northwest China, compounded by periodic cold events during the Last Glacial Period, has collectively reshaped the evolutionary trajectory of *L. racemosus* (Guo et al.,1999; Xu et al., 2010, Xu et al., 2010). Intense climatic fluctuations led to a sharp reduction in river flow and increasing habitat fragmentation (Huang et al., 2023), imposing strong natural selection pressures on the species while simultaneously restructuring the spatial configuration of its habitat through geomorphological changes (Zhang and Sun, 2011). Previous studies have shown that glacial activity can reshape river networks, thereby triggering genetic differentiation in species (Zhang et al., 2008; Zhang et al., 2011; Zhao et al., 2013; Panin et al., 2020). With the repeated advances and retreats of glaciers during glacial periods, the periodic formation and disappearance of ephemeral river networks may have provided a unique dynamic of dispersal-isolation for *L. racemosus*. These river networks acted as corridors for gene flow, during humid periods, whereas they transformed into geographical barriers during arid, cold periods, reinforcing local adaptation. The onset of vegetation succession during this interval was closely synchronized with the severe climatic changes, showing no apparent lag effect (Xu et al., 2023). Therefore, the intense climatic fluctuations of the Quaternary glacial periods, coupled with persistent aridification, likely represent key factors driving the population genetic differentiation of *L. racemosus*.

The interplay between isolation by distance (IBD) and isolation by environment (IBE) highlights the complex nature of genetic divergence in natural environments, where geographic isolation restricts gene flow while environmental variability exacerbates differentiation by creating that favor specific genetic traits (Tian et al., 2024; Ma et al., 2025). Gradient forest (GF) and redundancy analysis (RAD) identified precipitation and temperature as the primary environmental variables influencing genetic variation and structure in *L. racemosus*, while soil factors played a minimal role. This pattern is likely related to the species’ ecological preference for dune habitats characterized by limited rainfall and pronounced environmental selectivity (Dong et al., 1985). The restricted precipitation and fluctuating temperatures significantly affect both the distribution and genetic characteristics of *L. racemosus*. Our study is in high agreement with previous studies, for instance, genetic divergence and adaptive divergence in populations of the *Caragana genus* have been significantly associated with variations in precipitation (Yang et al., 2013). Additionally, changes in temperature and precipitation have been linked to adaptive differentiation in *Fagus sylvatica* and *Microhyla fissipes* in Europe (Postolache et al., 2021; Jin et al., 2022). Similarly, the genetic variation and adaptive evolution of *Actinidia eriantha* are influenced by both precipitation and solar radiation (Jiang et al., 2025). In natural environments, climate variability alters resource distribution and selection pressures, while geomorphic evolution shapes genetic structures by influencing isolation and migration corridors (Liu et al., 2017b; Harris et al., 2018).

### 4.3 Conservation recommendations

Studies on population genetic diversity and genetic structure in relation to environmental factors are fundamental for developing effective conservation strategies and rationally utilizing genetic resources (Li et al., 2025). *L. racemosus*, a wild relative of wheat, has been designated as a Category III protected plant in Xinjiang, China (Yin et al., 2006). Given the low genetic diversity of *L. racemosus* and its significant shelter vulnerability to environmental factors, we propose establishing protected areas within its current distribution range, including the Irtysh-Ulungur dual-basin and Kalamiris Mountain. This conservation measure aims to safeguard both the species and its critical habitats while simultaneously mitigating human-induced disturbances. Additionally, systematically collecting seeds and rhizome materials from geographically distinct populations will facilitate the establishment of cryopreserved seed banks and *ex situ* living gene banks (via *in situ* cultivation). These measures will ensure the effective preservation of existing genetic resources, safeguarding the evolutionary potential of *L. racemosus* in the face of environmental challenges.

## 5 Conclusion

In summary, this study provides a comprehensive analysis of the interplay between genetic structure and natural landscape patterns in *L. racemosus*. It demonstrates that geographic distance, environmental factors, and their interactions play critical roles in driving genetic divergence. The findings indicate that the river system is the primary factor influencing genetic divergence among lineages, while climatic variables such as precipitation and temperature also significantly shape genetic variation and structure. These results enhance our understanding of how genetic structure correlates with natural landscape patterns and provide theoretical support for the conservation and genetic resource utilization of *L. racemosus.*

## Data Availability

The data presented in this study are deposited in the NGDC repository, accession number CRA023768.
