# Supplementary material for: Drivers of intraspecific genetic differentiation of a wheat’s wild relative Leymus racemosus: roles of isolation by distance and environmental factors
Source: Front Plant Sci. 2025 Nov 18;16:1675027. doi: 10.3389/fpls.2025.1675027 (PMC12673403; doi:10.3389/fpls.2025.1675027)
Supplement: Supplementary file 1 [file DataSheet1.docx]

Supplementary Material

# Supplementary Figures and Tables

## Supplementary Tables

**Supplementary Table S1.** Experimental materials and sources

| Population | Latitude (N) | Longitude (E) | Altitude (m) |
| --- | --- | --- | --- |
| XB1 | 47°14′ | 88°26′ | 576 |
| BJ2 | 47°23′ | 87°50′ | 454 |
| KL3 | 47°25′ | 87°47′ | 454 |
| XH4 | 47°26′ | 87°29′ | 443 |
| KE5 | 47°38′ | 86°59′ | 429 |
| GL6 | 47°46′ | 86°46′ | 433 |
| BS7 | 47°58′ | 86°49′ | 448 |
| KM8 | 47°58′ | 86°33′ | 490 |
| KB9 | 47°59′ | 86°23′ | 443 |
| DL10 | 47°54′ | 86°17′ | 411 |
| BQ11 | 47°58′ | 85°55′ | 446 |
| BT12 | 48°20′ | 85°40′ | 381 |
| SS13 | 48°14′ | 85°40′ | 432 |
| HB14 | 47°56′ | 85°34′ | 381 |
| JK15 | 47°40′ | 85°37′ | 570 |
| HJ16 | 47°90′ | 87°34′ | 453 |
| WL17 | 47°70′ | 87°18′ | 434 |
| MG18 | 47°40′ | 87°29′ | 478 |
| KK19 | 45°14′ | 89°4′ | 940 |
| MM20 | 45°20′ | 89°15′ | 964 |
| TM21 | 46°28′ | 89°3′ | 762 |
| TS22 | 46°27′ | 88°34′ | 623 |
| FH23 | 46°32′ | 88°16′ | 590 |
| CZ24 | 46°42′ | 87°45′ | 518 |
| SD25 | 46°55′ | 87°45′ | 481 |
| AK26 | 47°34′ | 85°52′ | 734 |
| WT27 | 47°42′ | 86°3′ | 558 |

**Supplementary** **Table S2.** Statistical summary of dd-RAD sequencing data from 135 individuals of *Leymus racemosus* in 27 populations

| Population | Sample | Raw_Reads | Total_Bases(bp) | GC(%) | Q20(%) | Q30(%) | Mapping Rate | Sequencing Depth |
| --- | --- | --- | --- | --- | --- | --- | --- | --- |
| XB1 | XB101 | 35,111,900 | 5,056,113,600 | 45.25 | 98.97 | 96.89 | 98.61% | 0.9944 |
|  | XB102 | 35,111,900 | 5,056,113,600 | 44.74 | 99.03 | 97.06 | 98.72% | 0.9970 |
|  | XB103 | 35,111,900 | 5,056,113,600 | 42.94 | 99.29 | 97.78 | 98.85% | 0.9988 |
|  | XB104 | 35,111,900 | 5,056,113,600 | 43.71 | 99.18 | 97.48 | 98.90% | 0.9981 |
|  | XB105 | 35,111,900 | 5,056,113,600 | 44.94 | 98.87 | 96.77 | 98.79% | 0.9850 |
| BJ2 | BJ201 | 35,111,900 | 5,056,113,600 | 44.82 | 99.05 | 97.12 | 98.73% | 0.9969 |
|  | BJ202 | 35,111,900 | 5,056,113,600 | 43.94 | 98.81 | 96.62 | 98.91% | 0.9928 |
|  | BJ203 | 35,111,900 | 5,056,113,600 | 45.14 | 98.96 | 96.88 | 98.55% | 0.9929 |
|  | BJ204 | 35,111,900 | 5,056,113,600 | 43.92 | 98.81 | 96.63 | 98.86% | 0.9904 |
|  | BJ205 | 35,111,900 | 5,056,113,600 | 44.84 | 98.98 | 96.93 | 98.61% | 0.9935 |
| KL3 | KL301 | 35,111,900 | 5,056,113,600 | 45.62 | 99.01 | 97.00 | 98.64% | 0.9958 |
|  | KL302 | 35,111,900 | 5,056,113,600 | 44.89 | 98.98 | 96.94 | 98.65% | 0.9939 |
|  | KL303 | 33,602,706 | 4,838,789,664 | 45.23 | 98.94 | 96.82 | 98.53% | 0.9682 |
|  | KL304 | 35,111,900 | 5,056,113,600 | 45.93 | 98.89 | 96.65 | 98.62% | 0.9950 |
|  | KL305 | 35,111,900 | 5,056,113,600 | 44.89 | 99.03 | 97.08 | 98.51% | 1.0312 |
| XH4 | XH401 | 35,111,900 | 5,056,113,600 | 45.33 | 98.96 | 96.91 | 98.68% | 0.9954 |
|  | XH402 | 35,111,900 | 5,056,113,600 | 44.89 | 99.13 | 97.29 | 98.73% | 0.9980 |
|  | XH403 | 35,111,900 | 5,056,113,600 | 44.34 | 98.91 | 96.83 | 98.81% | 1.0374 |
|  | XH404 | 35,111,900 | 5,056,113,600 | 43.89 | 98.86 | 96.72 | 98.68% | 0.9875 |
|  | XH405 | 35,111,900 | 5,056,113,600 | 44.25 | 98.85 | 96.70 | 98.83% | 0.9888 |
| KE5 | KE501 | 35,111,900 | 5,056,113,600 | 44.16 | 98.82 | 96.66 | 98.59% | 0.9975 |
|  | KE502 | 35,111,900 | 5,056,113,600 | 44.11 | 98.83 | 96.64 | 98.81% | 0.9891 |
|  | KE503 | 35,111,900 | 5,056,113,600 | 45.27 | 98.96 | 96.89 | 98.57% | 0.9949 |
|  | KE504 | 35,111,900 | 5,056,113,600 | 44.21 | 98.82 | 96.64 | 98.72% | 0.9843 |
|  | KE505 | 35,111,900 | 5,056,113,600 | 44.59 | 99.03 | 97.08 | 98.89% | 0.998 |
| GL6 | GL601 | 35,111,900 | 5,056,113,600 | 45.19 | 99.03 | 97.02 | 98.66% | 0.9965 |
|  | GL602 | 35,111,900 | 5,056,113,600 | 44.63 | 98.99 | 96.93 | 98.94% | 1.0329 |
|  | GL603 | 35,111,900 | 5,056,113,600 | 44.81 | 99.01 | 96.99 | 98.71% | 0.9975 |
|  | GL604 | 35,111,900 | 5,056,113,600 | 45.07 | 98.95 | 96.85 | 98.60% | 0.9957 |
|  | GL605 | 35,111,900 | 5,056,113,600 | 44.63 | 98.93 | 96.78 | 98.56% | 0.9954 |
| BS7 | BS701 | 35,111,900 | 5,056,113,600 | 45.52 | 98.93 | 96.79 | 98.50% | 0.9944 |
|  | BS702 | 35,111,900 | 5,056,113,600 | 44.87 | 98.93 | 96.77 | 98.50% | 0.9946 |
|  | BS703 | 35,111,900 | 5,056,113,600 | 45.10 | 98.98 | 96.93 | 98.45% | 0.9946 |
|  | BS704 | 35,111,900 | 5,056,113,600 | 45.39 | 98.96 | 96.87 | 98.48% | 0.9925 |
|  | BS705 | 35,111,900 | 5,056,113,600 | 44.68 | 99.01 | 97.00 | 98.62% | 0.9939 |

Continued S2

| Population | Sample | Raw_Reads | Total_Bases(bp) | GC(%) | Q20(%) | Q30(%) | Mapping Rate | Sequencing Depth |
| --- | --- | --- | --- | --- | --- | --- | --- | --- |
| KM8 | KM801 | 35,111,900 | 5,056,113,600 | 44.37 | 98.81 | 96.61 | 98.53% | 0.9872 |
|  | KM802 | 35,111,900 | 5,056,113,600 | 43.76 | 98.86 | 96.74 | 98.81% | 0.9933 |
|  | KM803 | 35,111,900 | 5,056,113,600 | 43.45 | 98.92 | 96.87 | 99.00% | 0.9903 |
|  | KM804 | 35,111,900 | 5,056,113,600 | 44.33 | 98.85 | 96.73 | 98.75% | 0.9919 |
|  | KM805 | 35,111,900 | 5,056,113,600 | 43.90 | 98.82 | 96.63 | 98.35% | 1.0156 |
| KB9 | KB901 | 35,111,900 | 5,056,113,600 | 44.61 | 98.78 | 96.56 | 98.22% | 0.5877 |
|  | KB902 | 35,111,900 | 5,056,113,600 | 43.95 | 98.83 | 96.67 | 98.65% | 1.0234 |
|  | KB903 | 35,111,900 | 5,056,113,600 | 44.43 | 98.73 | 96.45 | 98.34% | 0.9482 |
|  | KB904 | 35,111,900 | 5,056,113,600 | 44.13 | 98.86 | 96.74 | 98.82% | 0.9877 |
|  | KB905 | 35,111,900 | 5,056,113,600 | 44.96 | 98.78 | 96.55 | 98.74% | 0.9920 |
| DL10 | DL1001 | 35,111,900 | 5,056,113,600 | 44.71 | 98.83 | 96.68 | 98.79% | 0.9845 |
|  | DL1002 | 35,111,900 | 5,056,113,600 | 44.90 | 98.76 | 96.49 | 98.92% | 1.0341 |
|  | DL1003 | 35,111,900 | 5,056,113,600 | 44.97 | 98.76 | 96.53 | 98.90% | 0.9910 |
|  | DL1004 | 35,111,900 | 5,056,113,600 | 43.65 | 98.88 | 96.78 | 98.83% | 0.9858 |
|  | DL1005 | 35,111,900 | 5,056,113,600 | 46.78 | 98.78 | 96.56 | 98.75% | 0.9876 |
| BQ11 | BQ1101 | 34,703,516 | 4,997,306,304 | 44.25 | 98.75 | 96.52 | 98.89% | 0.9860 |
|  | BQ1102 | 35,111,900 | 5,056,113,600 | 45.03 | 98.92 | 96.88 | 98.73% | 1.0567 |
|  | BQ1103 | 35,111,900 | 5,056,113,600 | 43.93 | 98.85 | 96.71 | 98.85% | 0.9946 |
|  | BQ1104 | 31,353,684 | 4,514,930,496 | 44.07 | 99.04 | 97.12 | 98.70% | 0.9328 |
|  | BQ1105 | 35,083,676 | 5,052,049,344 | 43.68 | 98.96 | 97.00 | 98.88% | 0.9941 |
| BT12 | BT1201 | 35,111,900 | 5,056,113,600 | 44.52 | 98.89 | 96.85 | 98.72% | 0.9830 |
|  | BT1202 | 35,111,900 | 5,056,113,600 | 43.82 | 98.84 | 96.68 | 98.88% | 0.9947 |
|  | BT1203 | 35,111,900 | 5,056,113,600 | 44.01 | 99.02 | 97.16 | 98.86% | 0.9889 |
|  | BT1204 | 35,111,900 | 5,056,113,600 | 43.52 | 98.82 | 96.66 | 98.97% | 0.9953 |
|  | BT1205 | 35,111,900 | 5,056,113,600 | 44.40 | 98.97 | 97.01 | 98.80% | 0.9883 |
| SS13 | SS1301 | 35,111,900 | 5,056,113,600 | 43.68 | 98.88 | 96.79 | 98.70% | 0.9841 |
|  | SS1302 | 35,111,900 | 5,056,113,600 | 43.39 | 98.81 | 96.63 | 98.86% | 1.0056 |
|  | SS1303 | 35,111,900 | 5,056,113,600 | 43.60 | 98.81 | 96.61 | 98.73% | 0.9932 |
|  | SS1304 | 33,297,390 | 4,794,824,160 | 43.98 | 99.06 | 97.20 | 98.60% | 0.9641 |
|  | SS1305 | 35,111,900 | 5,056,113,600 | 44.27 | 99.06 | 97.19 | 98.69% | 0.9971 |
| HB14 | HB1401 | 35,111,900 | 5,056,113,600 | 43.53 | 98.80 | 96.62 | 98.89% | 1.0347 |
|  | HB1402 | 35,111,900 | 5,056,113,600 | 43.81 | 98.87 | 96.78 | 98.93% | 0.9888 |
|  | HB1403 | 35,111,900 | 5,056,113,600 | 43.23 | 98.80 | 96.60 | 98.91% | 0.9957 |
|  | HB1404 | 35,111,900 | 5,056,113,600 | 43.83 | 98.77 | 96.55 | 98.90% | 0.9948 |
|  | HB1405 | 35,111,900 | 5,056,113,600 | 44.12 | 98.84 | 96.72 | 98.93% | 0.9889 |
| JK15 | JK1501 | 35,111,900 | 5,056,113,600 | 44.22 | 98.85 | 96.71 | 98.73% | 0.9954 |
|  | JK1502 | 35,111,900 | 5,056,113,600 | 45.32 | 98.78 | 96.55 | 98.81% | 0.9915 |
|  | JK1503 | 35,111,900 | 5,056,113,600 | 44.40 | 98.88 | 96.78 | 98.64% | 0.9848 |
|  | JK1504 | 35,111,900 | 5,056,113,600 | 43.93 | 98.79 | 96.58 | 98.86% | 0.9837 |
|  | JK1505 | 35,111,900 | 5,056,113,600 | 43.63 | 98.81 | 96.63 | 98.80% | 0.9939 |

Continued S2

| Population | Sample | Raw_Reads | Total_Bases(bp) | GC(%) | Q20(%) | Q30(%) | Mapping Rate | Sequencing Depth |
| --- | --- | --- | --- | --- | --- | --- | --- | --- |
| HJ16 | HJ1601 | 35,111,900 | 5,056,113,600 | 43.24 | 98.85 | 96.72 | 98.94% | 0.9961 |
|  | HJ1602 | 35,111,900 | 5,056,113,600 | 43.45 | 98.81 | 96.62 | 98.90% | 0.9972 |
|  | HJ1603 | 35,111,900 | 5,056,113,600 | 43.70 | 98.79 | 96.58 | 98.78% | 1.0079 |
|  | HJ1604 | 35,111,900 | 5,056,113,600 | 43.11 | 98.84 | 96.72 | 98.89% | 0.9973 |
|  | HJ1605 | 35,111,900 | 5,056,113,600 | 42.70 | 98.91 | 96.85 | 98.45% | 1.0089 |
| WL17 | WL1701 | 35,111,900 | 5,056,113,600 | 44.85 | 98.80 | 96.61 | 98.86% | 1.0142 |
|  | WL1702 | 35,111,900 | 5,056,113,600 | 45.31 | 98.80 | 96.62 | 98.80% | 0.9897 |
|  | WL1703 | 35,111,900 | 5,056,113,600 | 42.51 | 98.88 | 96.78 | 98.92% | 0.9977 |
|  | WL1704 | 35,111,900 | 5,056,113,600 | 43.32 | 98.79 | 96.59 | 98.84% | 0.9958 |
|  | WL1705 | 35,111,900 | 5,056,113,600 | 42.56 | 98.84 | 96.71 | 98.32% | 0.9834 |
| MG18 | MG1801 | 34,017,268 | 4,898,486,592 | 44.36 | 98.82 | 96.62 | 98.66% | 0.9765 |
|  | MG1802 | 35,111,900 | 5,056,113,600 | 44.43 | 98.79 | 96.56 | 98.57% | 0.9904 |
|  | MG1803 | 35,111,900 | 5,056,113,600 | 44.54 | 98.76 | 96.50 | 98.60% | 0.9927 |
|  | MG1804 | 35,111,900 | 5,056,113,600 | 44.36 | 98.76 | 96.53 | 98.56% | 1.0047 |
|  | MG1805 | 35,111,900 | 5,056,113,600 | 44.73 | 98.74 | 96.46 | 98.92% | 1.0192 |
| KK19 | KK1901 | 35,111,900 | 5,056,113,600 | 46.12 | 98.82 | 96.66 | 98.65% | 0.9869 |
|  | KK1902 | 35,111,900 | 5,056,113,600 | 43.94 | 98.81 | 96.63 | 98.66% | 0.9903 |
|  | KK1903 | 35,111,900 | 5,056,113,600 | 42.88 | 98.83 | 96.67 | 98.87% | 0.9960 |
|  | KK1904 | 35,111,900 | 5,056,113,600 | 44.62 | 98.76 | 96.53 | 98.79% | 0.9936 |
|  | KK1905 | 35,111,900 | 5,056,113,600 | 43.83 | 98.72 | 96.44 | 98.85% | 0.9919 |
| MM20 | MM2001 | 31,916,376 | 4,595,958,144 | 44.70 | 99.03 | 97.12 | 98.46% | 0.9384 |
|  | MM2002 | 32,288,532 | 4,649,548,608 | 43.90 | 99.05 | 97.14 | 98.61% | 0.9481 |
|  | MM2003 | 35,111,900 | 5,056,113,600 | 45.91 | 98.93 | 96.81 | 98.44% | 0.9903 |
|  | MM2004 | 35,111,900 | 5,056,113,600 | 45.41 | 98.98 | 96.94 | 98.49% | 1.0357 |
|  | MM2005 | 35,111,900 | 5,056,113,600 | 46.19 | 98.91 | 96.75 | 98.26% | 0.9910 |
| TM21 | TM2101 | 35,111,900 | 5,056,113,600 | 44.70 | 98.10 | 95.41 | 98.80% | 0.9722 |
|  | TM2102 | 35,111,900 | 5,056,113,600 | 45.68 | 98.93 | 96.80 | 98.57% | 0.9930 |
|  | TM2103 | 35,111,900 | 5,056,113,600 | 46.40 | 99.02 | 96.96 | 98.49% | 1.0252 |
|  | TM2104 | 35,111,900 | 5,056,113,600 | 43.37 | 98.93 | 96.87 | 98.28% | 0.9879 |
|  | TM2105 | 35,111,900 | 5,056,113,600 | 45.20 | 97.62 | 94.44 | 98.46% | 0.9663 |
| TS22 | TS2201 | 35,111,900 | 5,056,113,600 | 47.06 | 98.03 | 95.18 | 98.34% | 0.9694 |
|  | TS2202 | 35,111,900 | 5,056,113,600 | 43.92 | 97.97 | 95.31 | 98.63% | 0.9722 |
|  | TS2203 | 35,111,900 | 5,056,113,600 | 45.87 | 98.97 | 96.82 | 98.48% | 0.9925 |
|  | TS2204 | 35,111,900 | 5,056,113,600 | 44.03 | 97.96 | 95.15 | 98.56% | 1.0379 |
|  | TS2205 | 35,111,900 | 5,056,113,600 | 45.52 | 99.02 | 97.04 | 98.87% | 1.0319 |
| FH23 | FH2301 | 33,027,518 | 4,755,962,592 | 45.77 | 97.86 | 95.10 | 98.54% | 0.9331 |
|  | FH2302 | 35,111,900 | 5,056,113,600 | 45.38 | 99.01 | 97.03 | 98.56% | 0.9930 |
|  | FH2303 | 35,111,900 | 5,056,113,600 | 45.54 | 98.94 | 96.84 | 98.52% | 1.0283 |
|  | FH2304 | 35,111,900 | 5,056,113,600 | 45.31 | 97.98 | 95.16 | 98.40% | 0.9721 |
|  | FH2305 | 35,111,900 | 5,056,113,600 | 45.24 | 98.09 | 95.38 | 98.59% | 1.0289 |

Continued S2

| Population | Sample | Raw_Reads | Total_Bases(bp) | GC(%) | Q20(%) | Q30(%) | Mapping Rate | Sequencing Depth |
| --- | --- | --- | --- | --- | --- | --- | --- | --- |
| CZ24 | CZ2401 | 35,111,900 | 5,056,113,600 | 46.66 | 98.13 | 95.33 | 97.79% | 0.9704 |
|  | CZ2402 | 35,111,900 | 5,056,113,600 | 45.95 | 98.90 | 96.61 | 98.33% | 0.9918 |
|  | CZ2403 | 35,111,900 | 5,056,113,600 | 45.68 | 98.32 | 95.72 | 98.46% | 0.9785 |
|  | CZ2404 | 35,111,900 | 5,056,113,600 | 46.41 | 97.69 | 94.40 | 98.24% | 0.9701 |
|  | CZ2405 | 35,111,900 | 5,056,113,600 | 47.23 | 97.92 | 94.76 | 98.08% | 0.9694 |
| SD25 | SD2501 | 35,111,900 | 5,056,113,600 | 46.86 | 98.00 | 95.13 | 98.32% | 0.9709 |
|  | SD2502 | 35,111,900 | 5,056,113,600 | 44.93 | 98.01 | 95.14 | 98.54% | 0.9780 |
|  | SD2503 | 35,111,900 | 5,056,113,600 | 45.70 | 98.40 | 95.84 | 98.29% | 0.9825 |
|  | SD2504 | 35,111,900 | 5,056,113,600 | 45.38 | 99.03 | 97.06 | 98.49% | 0.9954 |
|  | SD2505 | 35,111,900 | 5,056,113,600 | 44.34 | 99.06 | 97.16 | 98.65% | 0.9958 |
| AK26 | AK2601 | 35,111,900 | 5,056,113,600 | 43.53 | 99.08 | 97.22 | 98.78% | 1.0096 |
|  | AK2602 | 32,853,688 | 4,730,931,072 | 43.89 | 99.04 | 96.99 | 98.71% | 0.9594 |
|  | AK2603 | 35,111,900 | 5,056,113,600 | 43.82 | 99.10 | 97.27 | 98.65% | 0.9980 |
|  | AK2604 | 35,111,900 | 5,056,113,600 | 43.97 | 99.08 | 97.24 | 98.76% | 0.9990 |
|  | AK2605 | 35,111,900 | 5,056,113,600 | 43.61 | 99.10 | 97.28 | 98.78% | 0.9980 |
| WT27 | WT2701 | 35,111,900 | 5,056,113,600 | 43.76 | 99.03 | 97.06 | 98.60% | 0.9971 |
|  | WT2702 | 35,111,900 | 5,056,113,600 | 43.58 | 99.08 | 97.11 | 98.74% | 0.9984 |
|  | WT2703 | 35,111,900 | 5,056,113,600 | 43.91 | 99.02 | 96.89 | 98.59% | 1.0448 |
|  | WT2704 | 35,111,900 | 5,056,113,600 | 42.95 | 99.08 | 97.09 | 98.44% | 1.0503 |
|  | WT2705 | 35,111,900 | 5,056,113,600 | 43.00 | 99.07 | 97.12 | 98.78% | 0.9003 |

**Supplementary Table S3.** 16 Bioclimatic and Soil Data Sheets Corresponding to 27 Populations of *Leymus racemosus*

| Population | bio3 | bio6 | bio7 | bio8 | bio9 | bio10 | bio12 | bio15 | T_GRAVEL | T_REF | T_BULK | T_OC | T_CEC | T_BS | T_TEB | T_CACO3 |
| --- | --- | --- | --- | --- | --- | --- | --- | --- | --- | --- | --- | --- | --- | --- | --- | --- |
| XB1 | 23 | -192 | 499 | 235 | -54 | 235 | 124 | 28 | 9 | 1.4 | 1.37 | 1 | 55 | 93 | 14 | 0 |
| BJ2 | 23 | -189 | 501 | 241 | -50 | 241 | 146 | 32 | 9 | 1.4 | 1.37 | 1 | 55 | 93 | 14 | 0 |
| KL3 | 23 | -188 | 499 | 241 | -50 | 241 | 150 | 32 | 4 | 1.4 | 1.43 | 0.74 | 47 | 91 | 11 | 0 |
| XH4 | 23 | -187 | 498 | 241 | -111 | 241 | 158 | 40 | 9 | 1.4 | 1.37 | 1 | 55 | 93 | 14 | 0 |
| KE5 | 23 | -177 | 481 | 235 | -107 | 235 | 167 | 41 | 4 | 1.4 | 1.36 | 0.86 | 63 | 91 | 16.9 | 0.5 |
| GL6 | 23 | -172 | 471 | 231 | -104 | 231 | 185 | 40 | 4 | 1.41 | 1.4 | 0.99 | 48 | 62 | 8.4 | 0 |
| BS7 | 23 | -168 | 461 | 226 | -48 | 226 | 183 | 36 | 12 | 1.4 | 1.53 | 0.83 | 39 | 88 | 10 | 0 |
| KM8 | 23 | -165 | 458 | 226 | -101 | 226 | 190 | 34 | 4 | 1.39 | 1.28 | 1.65 | 45 | 87 | 9.8 | 0 |
| KB9 | 23 | -164 | 455 | 225 | -46 | 225 | 198 | 32 | 4 | 1.4 | 1.36 | 0.86 | 63 | 91 | 16.9 | 0.5 |
| DL10 | 23 | -163 | 458 | 229 | -99 | 229 | 195 | 31 | 9 | 1.4 | 1.37 | 1 | 55 | 93 | 14 | 0 |
| BQ11 | 23 | -158 | 450 | 228 | -43 | 228 | 224 | 29 | 4 | 1.39 | 1.28 | 1.65 | 45 | 87 | 9.8 | 0 |
| BT12 | 23 | -155 | 446 | 227 | -42 | 227 | 243 | 29 | 4 | 1.4 | 1.36 | 0.86 | 63 | 91 | 16.9 | 0.5 |
| SS13 | 23 | -158 | 442 | 220 | -46 | 220 | 279 | 32 | 14 | 1.41 | 1.37 | 0.75 | 45 | 100 | 26.8 | 15 |
| HB14 | 23 | -155 | 447 | 228 | -42 | 228 | 227 | 28 | 3 | 1.73 | 1.4 | 2.18 | 101 | 11 | 2.6 | 0 |
| JK15 | 23 | -167 | 451 | 219 | -105 | 219 | 219 | 31 | 14 | 1.41 | 1.37 | 0.75 | 45 | 100 | 26.8 | 15 |
| HJ16 | 23 | -195 | 514 | 247 | -116 | 247 | 123 | 43 | 16 | 1.41 | 1.24 | 2.13 | 80 | 100 | 28.8 | 6 |
| WL17 | 22 | -195 | 515 | 249 | -117 | 249 | 124 | 54 | 4 | 1.39 | 1.28 | 1.65 | 45 | 87 | 9.8 | 0 |
| MG18 | 23 | -199 | 516 | 246 | -119 | 246 | 132 | 49 | 12 | 1.4 | 1.53 | 0.83 | 39 | 88 | 10 | 0 |
| KK19 | 23 | -205 | 499 | 225 | -126 | 225 | 154 | 41 | 4 | 1.42 | 1.33 | 1.05 | 21 | 36 | 2.6 | 0 |
| MM20 | 23 | -208 | 498 | 221 | -129 | 221 | 174 | 46 | 4 | 1.42 | 1.33 | 1.05 | 21 | 36 | 2.6 | 0 |
| TM21 | 23 | -199 | 501 | 1 | -121 | 233 | 136 | 34 | 14 | 1.41 | 1.37 | 0.75 | 45 | 100 | 26.8 | 15 |

Continued S3

| Population | bio3 | bio6 | bio7 | bio8 | bio9 | bio10 | bio12 | bio15 | T_GRAVEL | T_REF | T_BULK | T_OC | T_CEC | T_BS | T_TEB | T_CACO3 |
| --- | --- | --- | --- | --- | --- | --- | --- | --- | --- | --- | --- | --- | --- | --- | --- | --- |
| TS22 | 23 | -201 | 515 | 243 | -121 | 243 | 109 | 38 | 9 | 1.4 | 1.37 | 1 | 55 | 93 | 14 | 0 |
| FH23 | 23 | -205 | 521 | 245 | -124 | 245 | 113 | 36 | 9 | 1.4 | 1.37 | 1 | 55 | 93 | 14 | 0 |
| CZ24 | 22 | -207 | 527 | 249 | -124 | 249 | 100 | 49 | 9 | 1.4 | 1.37 | 1 | 55 | 93 | 14 | 0 |
| SD25 | 22 | -201 | 522 | 249 | -50 | 249 | 109 | 41 | 16 | 1.41 | 1.24 | 2.13 | 80 | 100 | 28.8 | 6 |
| AK26 | 23 | -180 | 456 | 211 | -116 | 211 | 216 | 37 | 4 | 1.4 | 1.36 | 0.86 | 63 | 91 | 16.9 | 0.5 |
| WT27 | 23 | -171 | 458 | 222 | -51 | 222 | 190 | 29 | 4 | 1.71 | 1.49 | 0.4 | 35 | 93 | 3.3 | 0 |

Note. bio3. Isothermality; bio6. Minimum temperature of coldest month; bio7. Temperature annual range; bio8. Mean temperature of wettest quarter; bio9. Mean temperature of driest quarter; bio10. Mean temperature of warmest quarter; bio12. Annual precipitation; bio15. Precipitation seasonality; T_GRAVEL. Topsoil Gravel Content; T_REF. Topsoil Reference Value; T_BULK. Topsoil Bulk Density; T_OC. Topsoil Organic Carbon Content; T_CEC. Topsoil Cation Exchange Capacity; T_BS. Topsoil Base Saturation; T_TEB. Topsoil Total Exchangeable Bases; T_CACO3. Topsoil Calcium Carbonate Content.

**Supplementary Table S4.** Parameter Tab. of the Optimal Fitting Population ABC Model for *Leymus racemosus* lineages A and U

|  | Expectation | Median | Quantile_0.05 | Quantile_0.95 | Variance |
| --- | --- | --- | --- | --- | --- |
| td | 14751.1 | 10752.7 | 156 | 40822 | 107374000 |
| N1 | 169159 | 123604 | 17861.1 | 97832.2 | 596071000 |
| N2 | 120557 | 96638.7 | 47862.9 | 406831 | 11983000000 |
| NA | 271811 | 274908 | 93302.1 | 454391 | 7.839110000 |

**Supplementary Table S5.** The voting proportions, misclassification rates, and posterior probabilities (PPs) of three statistical models (Model 1-3) for the *Leymus racemosus* lineage A and U under different support scenarios were calculated based on the ABC random forest (ABC-RF) method.

| voting proportions | | | Classification error rate | PP of the best model |
| --- | --- | --- | --- | --- |
| Votes model 1 | Votes model 2 | Votes model 3 | 0.19 | 0.611 |
| 0.634 | 0.264 | 0.102 |  |  |

## Supplementary Figures


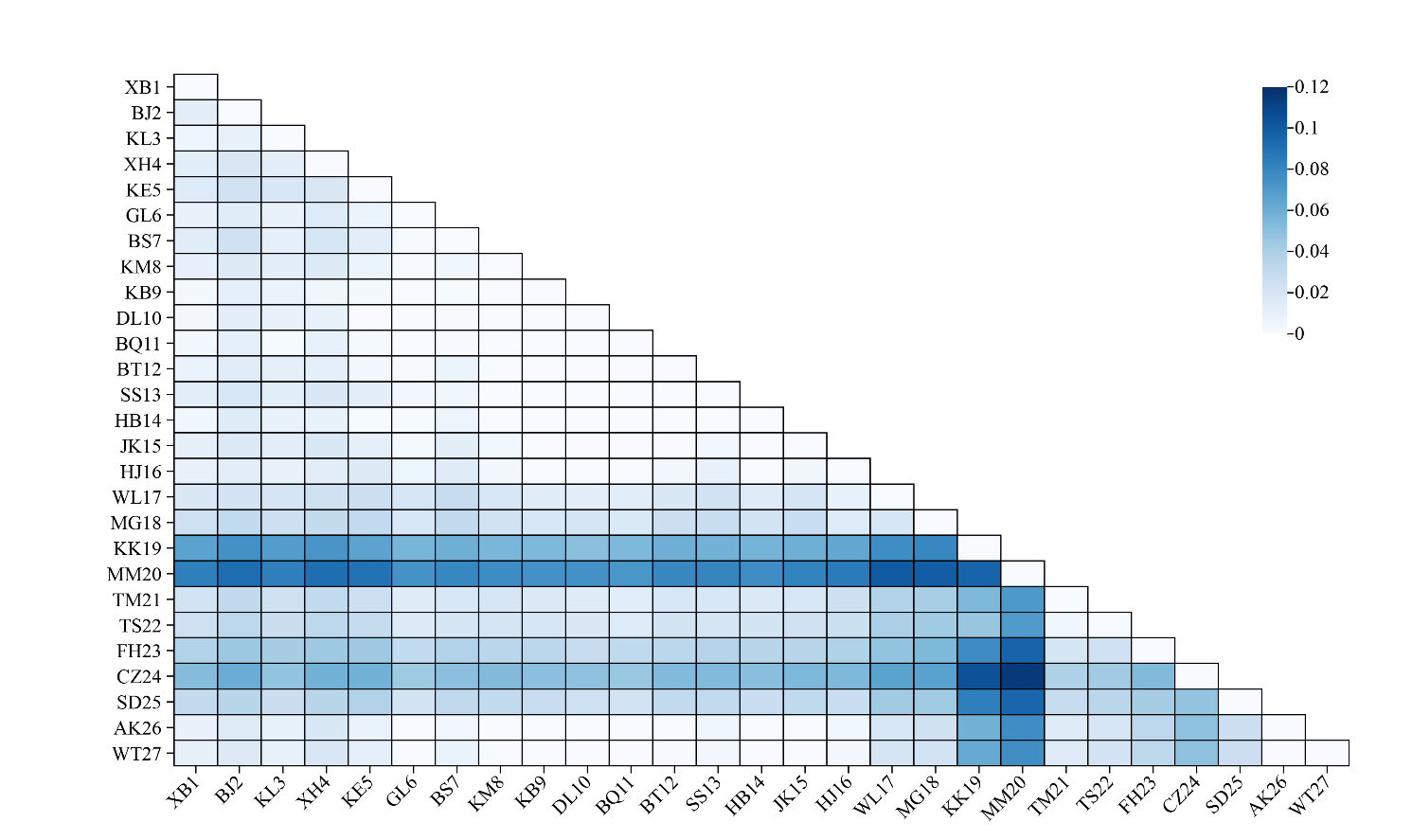


**Supplementary Figure S1.** Genetic differentiation (*F_ST_*) among different populations of *Leymus racemosus*


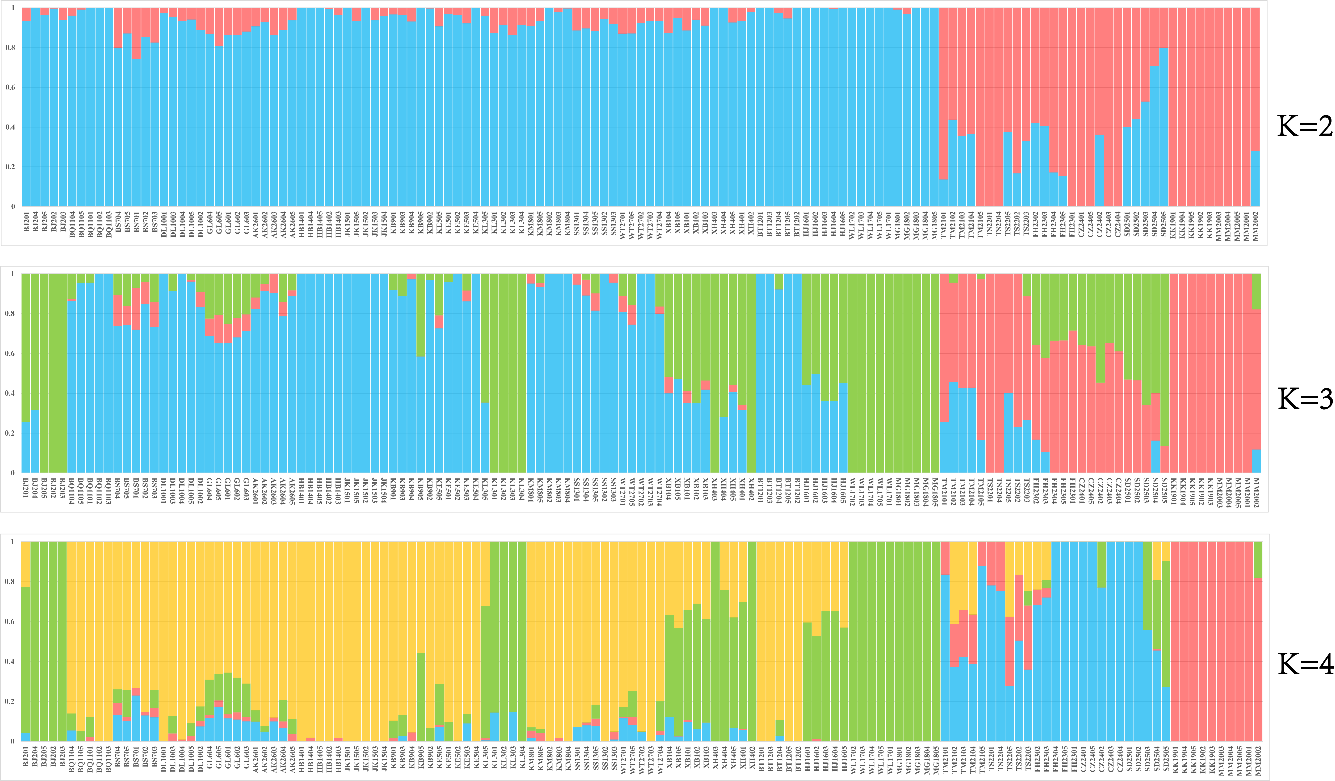


**Supplementary Figure S2.** Results of genetic clustering analysis of 27 *Leymus racemosus* populations based on ADMIXTURE software (K=2,3,4)


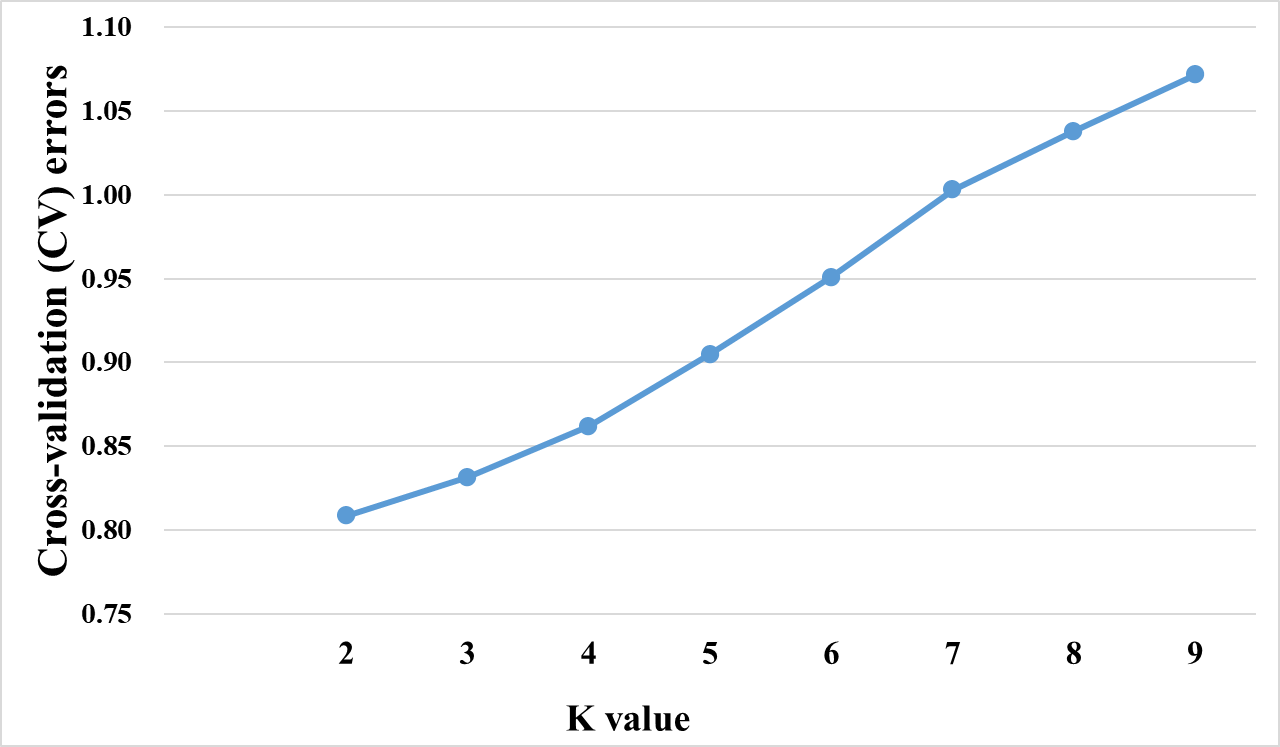


**Supplementary Figure S3.** The cross-validation error values based on the ADMIXTURE software vary with the K value (2-10). The plot shows the error value curve for 27 *Leymus racemosus* populations.


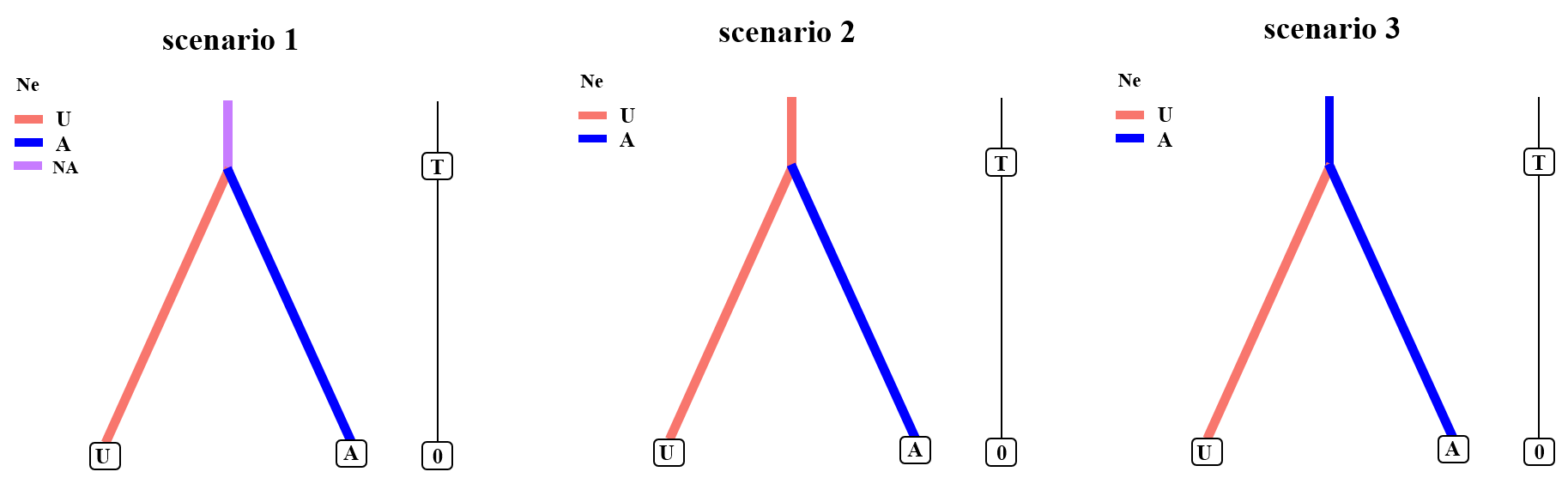


**Supplementary Figure S4.** The 3 scenarios of population history of two lineages in *Leymus racemosus* with *DIYABC*. Each branch of different colors represents a lineage. N1 and N2 represent the effective population size of the two lineages; NA represent the effective population size of ancestral population. T divergence times for the depicted event.
